# Artificial Neural Network-Assisted Classification of Hearing Prognosis of Sudden Sensorineural Hearing Loss With Vertigo

**DOI:** 10.1109/JTEHM.2023.3242339

**Published:** 2023-02-06

**Authors:** Sheng-Chiao Lin, Ming-Yee Lin, Bor-Hwang Kang, Yaoh-Shiang Lin, Yu-Hsi Liu, Chi-Yuan Yin, Po-Shing Lin, Che-Wei Lin

**Affiliations:** Department of Biomedical EngineeringCollege of Engineering, National Cheng Kung University 34912 Tainan 70101 Taiwan; Department of Otorhinolaryngology—Head and Neck SurgeryKaohsiung Veterans General Hospital 38024 Kaohsiung 813414 Taiwan; School of MedicineNational Defense Medical Center 71548 Taipei 11490 Taiwan; Department of Special EducationCollege of Education, National Kaohsiung Normal University 34873 Kaohsiung 80201 Taiwan; Medical Device Innovation CenterNational Cheng Kung University 34912 Tainan 70101 Taiwan

**Keywords:** Artificial intelligence, hearing prognosis, sudden sensorineural hearing loss, video head impulse test, wavelet coherence

## Abstract

This study aimed to determine the impact on hearing prognosis of the coherent frequency with high magnitude-squared wavelet coherence (MSWC) in video head impulse test (vHIT) among patients with sudden sensorineural hearing loss with vertigo (SSNHLV) undergoing high-dose steroid treatment. This study was a retrospective cohort study. SSNHLV patients treated at our referral center from December 2016 to December 2020 were examined. The cohort comprised 64 patients with SSNHLV undergoing high-dose steroid treatment. MSWC was measured by calculating the wavelet coherence analysis (WCA) at various frequencies from a vHIT. The hearing prognosis were analyzed using a multivariable Cox regression model and convolution neural network (CNN) of WCA. There were 64 patients with a male-to-female ratio of 1:1.67. The greater highest coherent frequency of the posterior semicircular canal (SCC) was associated with the complete recovery (CR) of hearing. After adjustment for other factors, the result remained robust (hazard ratio [HR] 2.11, 95% confidence interval [CI] 1.86-2.35). In the feature extraction with Resnet-50 and proceeding SVM in the horizontal image cropping style, the classification accuracy [STD] for (CR vs. partial + no recovery [PR + NR]), (over-sampling of CR vs. PR + NR), (extensive data extraction of CR vs. PR + NR), and (interpolation of time series of CR vs. PR + NR) were 83.6% [7.4], 92.1% [6.8], 88.9% [7.5], and 91.6% [6.4], respectively. The high coherent frequency of the posterior SCC was a significantly independent factor that was associated with good hearing prognosis in the patients who have SSNHLV. WCA may be provided with comprehensive ability in vestibulo-ocular reflex (VOR) evaluation. CNN could be utilized to classify WCA, predict treatment outcomes, and facilitate vHIT interpretation. Feature extraction in CNN with proceeding SVM and horizontal cropping style of wavelet coherence plot performed better accuracy and offered more stable model for hearing outcomes in patients with SSNHLV than pure CNN classification. Clinical and Translational Impact Statement—High coherent frequency in vHIT results in good hearing outcomes in SSNHLV and facilitates AI classification.

## I. Introduction

Sudden sensorineural hearing loss (SSNHL) is defined as sensorineural hearing loss of 30 dB or greater over at least three consecutive frequencies occurring within 72 hours [1]. Estimated incidence of SSNHL ranged from 11 to 77 per 100,000 people per year [2]. Throughout recent half-century, complete recovery (CR) of hearing still remained only 15.7-26% after steroid treatment [3], [4]. In SSNHL, 35.5% patients suffering from simultaneous vertigo with injured vestibular function, which was significantly associated with poor hearing outcome [5]. To objectively evaluate the vestibular function in patients suffering from vertigo, the video head impulse test (vHIT) was a new instrument to evaluate vestibular function with vestibulo-ocular reflex (VOR) function, where the ability to maintain images on the retina and in the center of visual field during highly-accelerated head movement by driving the eye toward to the contralateral side of the head movement was tested [6]. The vHIT defined the vestibulopathy as decreased VOR gain by time series velocity data of paired head and eye motions [7]. In addition to the VOR gain, saccades during and after the head impulse were also associated with the extent of vestibular damage and compensation [8], [9]. Research about the vestibular parameter significantly determining treatment response in patients of SSNHL with vertigo (SSNHLV) was still scarce [10], but some promising studies revealed the abnormal VOR gain of the posterior semicircular canal (SCC) was significantly associated with the poor hearing prognosis in patients with SSNHL [11], [12], [13]. Recently, the recovery process of human VOR function after vestibular injury called as VOR adaptation has been proved to be frequency-selective [14], [15]. The vHIT is currently the updated tool of vestibular exam in clinical application, but the study utilizing the time-frequency analysis of vHIT is still lacking. Therefore, in light of the time-frequency analysis between neural signals and causal response of body muscles, the coherence was considered to be the appropriate approach to measure the overall synchrony of the VOR and may provide the greater power of prognosis prediction in patients with SSNHLV than the current time series analysis [16].

In recent years, artificial intelligence has exerted its potential to reform health care delivery in otolaryngology field [17], [18]. For clinical physicians in vertigo clinics, the judgement of various vestibular exams was complicated and time-consuming. The artificial neural network (ANN) has been utilized to detect time-frequency plots in the pulse audiogram, the electroencephalography, and the gait analysis [19], [20], [21]. In this study, we took advantage of the ANN-assisted image classification of coherence plots to predict the hearing prognosis in patients with SSNHLV, facilitate clinical judgement, and develop the foundation of smart healthcare application in otoneurology.

## II. Materials and Methods

### A. Ethical Considerations

This study was approved by the Institutional Review Board of Kaohsiung Veterans General Hospital, Taiwan**(**IRB: KSVGH21-CT4-04). The requirement for informed consent was waived because all identifying information was removed from the dataset before analysis.

### B. Patients and Study Design

We retrospectively reviewed the electronic medical records of patients with SSNHLV as their primary treatment at a tertiary medical center in Taiwan from December 2016 to December 2020. We excluded patients with hearing loss without treatment within 14 days, pre-existing hearing loss in the contra-lesioned ear over 25 dB HL, retrocochlear pathology, other otologic diseases involving hearing loss, or anterior inferior cerebellar artery infarction on magnetic resonance imaging. After the exclusions, 64 patients were enrolled in the study.

Patients with SSNHL were treated with our standard protocol. Oral prednisolone (1mg/kg/day with maximal dose 60mg/day) or intravenous dexamethasone (5mg/ml b.i.d.) was given for 14 days including 3–4 days course of tapering, and salvage intratympanic dexamethasone injection (5mg/ml) was performed twice per week during 2 weeks for those whose hearing recovery did not reach complete recovery.

### C. Data Collection

Pure tone audiometry and word recognition score were performed at the first visit at our clinic and followed weekly during treatment course and 2 months after treatment. The pure tone audiometry, the highly accurate exam with simulated single-test root-mean-square error about 2.7 dB HL, was the gold standard evaluation to show the hearing threshold of the patient by dB HL [22]. The word recognition score presented the proportion of correctly repeated words by the patients.

The hearing recovery was defined by American Academy of Otolaryngology-Head and Neck Surgery in 2019, as CR (return to within 10 dB HL of the unaffected ear in pure tone audiometry and recovery of word recognition scores to within 5% to 10% of the unaffected ear), no recovery (NR, anything less than a 10–dB HL improvement in pure tone audiometry), and partial recovery (PR, recovery levels between CR and NR) [1].

Vestibular laboratory data were also collected within 2 weeks, including vHIT, cervical and ocular vestibular myogenic evoked potentials (c- and o-VEMP), caloric test, and posturography.

### D. vHIT

The vHIT was performed with ICS impulse^®^ (Otometrics A/S, Taastrup, Denmark) according to the protocol proposed by Halmagyi et al. [7] The patient’s head was passively rotated by examiner suddenly and unpredictably in the plane of each SCC pairs within about 5° in about 100ms, and the corresponding ratio of the eye velocities to the head peak velocities were counted as VOR gain. The ICS impulse^®^ automatically reported the head and eye velocities for 0.7s, and the onset of head rotation was always relocated at 0.1s in the timeline to make sure the complete record of the VOR impulse (0.1-0.2s in the timeline) and the following saccades (0.2-0.7s in the timeline). At least 10 times exams were repeated for each six SCCs, and the mean VOR gain and the gain asymmetry value within paired SCCs were recorded and regarded as abnormal compared with the mean and standard deviation of the group of normal healthy subjects in our hospital. The asymmetry values were counted specifically according to the planes tested as the right horizontal to the left horizontal, the left anterior to the right posterior, or the right anterior to the left posterior in the way of normalized relative gain asymmetry [23], [24].

### E. Wavelet Coherence Plot and Magnitude-Squared Wavelet Coherence

The analytic Morlet wavelet transform was utilized to analyze the time series data comprising nonstationary power at diverse frequencies [25], [26]. Cross wavelet power of paired head and eye velocities during vHIT was defined as wavelet transformation of the autocorrelation function and showed area with high coherent power in time-frequency domain. The wavelet coherence analysis (WCA) of two time series data was denoted and adapted from Grinsted et al. [27] as

\begin{equation*} {\boldsymbol {R}}_{n}^{2}\left ({s }\right)=\frac {\left |{ {S}\left ({s^{-1}{W}_{n}^{XY}\left ({s }\right) }\right) }\right |^{2}}{S\left ({s^{-1}\left |{ {W}_{n}^{X}\left ({s }\right) }\right |^{2} }\right).{}{S}\left ({s^{-1}\left |{ {W}_{n}^{Y}\left ({s }\right) }\right |^{2} }\right)}, \tag{1}\end{equation*}

where $S$ acted as the smoothing operator as

\begin{equation*} {\boldsymbol { S}}\left ({{\boldsymbol {W}} }\right)={\boldsymbol {S}}_{{\text {scale}}}\left ({{\boldsymbol {S}}_{{\text {time}}}\left ({{\boldsymbol {W}}_{n}(s) }\right) }\right) \tag{2}\end{equation*}

The wavelet scale $s$ indicated frequency as per wavelet notation. For head and eye velocities annotated as $X$ and $Y$, the ${\boldsymbol {W}}_{n}^{X}\left ({s }\right)$ and ${\boldsymbol {W}}_{n}^{Y}\left ({s }\right)$ represented their wavelet transformation, and the ${\boldsymbol {W}}_{n}^{XY}\left ({s }\right)$ was the cross-wavelet spectrum equal to ${\boldsymbol {W}}_{n}^{X}\left ({s }\right){\boldsymbol {W}}_{n}^{Y^{\ast }}\left ({s }\right)$. $S_{\mathrm {scale}}$ and $S_{{\text {time}{ }}}$ indicated smoothing process along the wavelet scale and time axes, and the ${\boldsymbol {R}}{n}^{2}\left ({s }\right)$ was the magnitude-squared wavelet coherence (MSWC), representing the estimated cross-spectral high common power.

The WCA was applied to every repeated exam for each SCCs, and the MSWC of every frequency was calculated. The coherent frequency in our study was defined as the highest frequency comprising MSWC 0.9 over entire exam (0-0.7s in the timeline) evaluated from the lowest frequency in WCA. Since the velocity of head rotation was fixed in vHIT, the coherent frequency of WCA was solely affected by the responsive eye velocities of VOR impulse and following saccades.

### F. Data Augmentation and Preprocessing

**FIGURE 1.**
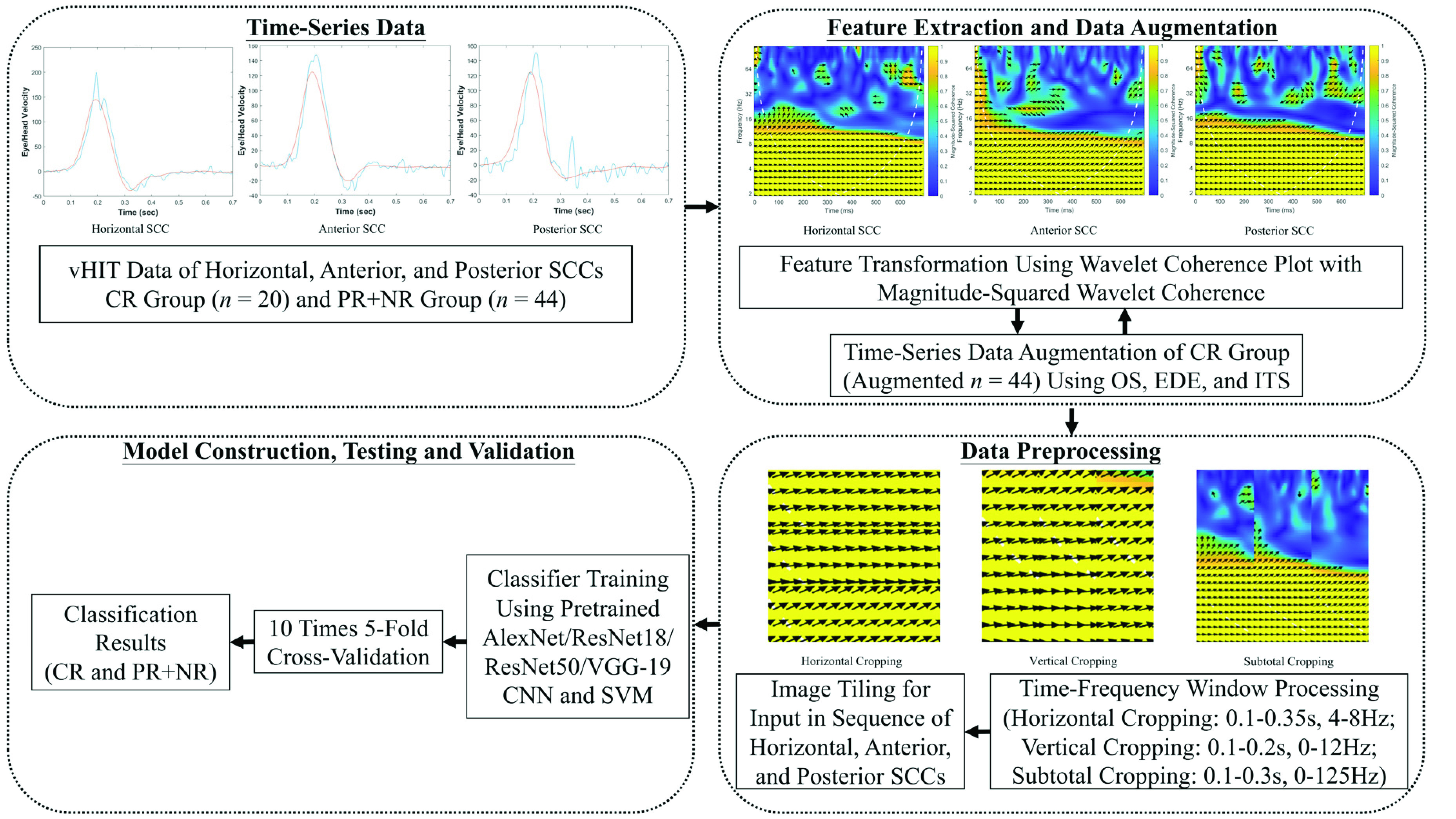
Flowchart of the proposed hearing outcomes prediction algorithm using wavelet coherence plot as the feature transformation and ANN models.

The algorithm of data preprocessing was made by MATLAB R2019b (MathWorks, Inc., Natick, MA, USA) and showed in Figure 1.

For further ANN image classification, only one power spectrum wavelet coherence plot comprising the median of the coherent frequencies among repeated exams for each SCCs was chosen. For each subject, there were three specific wavelet coherence plots representative for the horizontal, the anterior, and the posterior SCCs after the selection.

The methods of augmentation across imbalanced class of CR ( $n =20$) to achieve equal sample numbers as in class of PR + NR ( $n =44$) included simple over-sampling (OS), extensive data extraction (EDE), and interpolation of time series (ITS). In the method of OS, the time series data of paired head and eye velocities of the representative wavelet coherence plots previously chosen for each SCC were copied randomly in class of CR. In the method of EDE, among repeated vHITs in each SCC, the time series data lines with the closest coherent frequency to the highest coherent frequency of representative wavelet coherence plots previously chosen were extra extracted. In the method of ITS, the time series data lines of the representative wavelet coherence plots previously chosen for each SCC were interpolated with random weights between different subjects in class of CR to create augmented samples [28]. The final samples of augmented CR and PR + NR were $n =44$ equally after OS, EDE, or ITS. WCA for all augmented time series data were performed for further ANN classification.

In order to fit the formal protocol of pixel in convolutional neural network (CNN), the time and frequency windows were processed to 0.1-0.35s, 0.1-0.2s, and 0.1-0.3s started from the exam and 4-8Hz, 0-12Hz, and 0-125Hz for horizontal, vertical, and subtotal styles for image cropping of wavelet coherence plots. The period of passive head motion and median frequency of the highest coherent frequency in each SCCs were contained in these windows to make sure adequate principal component analysis of CNN classification in all three styles of image cropping. Finally, one image per patient including all three SCCs of the ipsilesional side was tiled from the left to the right or the superior to the inferior in the sequence of the horizontal, the anterior, and the posterior SCCs according to the cropping styles.

### G. Convolutional Neural Network and K-Fold Cross-Validation

Transfer learning in AlexNet, ResNet-18, and VGG-19 and feature extraction in AlexNet, ResNet-50, and VGG-19 with proceeding support vector machine (SVM) were employed by using the MATLAB R2019b Deep Learning Toolbox™. The CNN input was the tiled image containing time–frequency power spectrogram of the wavelet coherence between eye and head velocities in vHIT.

In this study, data were stratified before partition into five-folds to establish excellent representative of the entire sample in each fold. The repeated five-fold cross-validation method was applied for iterations of training and testing.

### H. Statistical Analysis

All statistical analyses were performed using SPSS ver. 22 (SPSS, Inc., Chicago, IL, USA). Continuous variables were analyzed using one-way analysis of variance (ANOVA), and categorical variables were compared using Pearson’s chi-squared test or Fisher’s exact test. The independent variables were determined through multivariate analysis using the forward stepwise method. A two-sided $p < 0.05$ was regarded as statistically significant.

## III. Results

Ultimately, 64 patients with SSNHLV were included in the analysis (mean [STD] age, 53 [15] years; 24 males [37.5%], and 40 females [62.5%]). The demographic characteristics of the cohort are summarized in Table 1. Among these patients, 31.3% got CR ( $n =20$), 28.1% got PR ( $n =18$), and 40.6% got NR ( $n =26$). The mean VOR gain was 0.98, 0.96, and 0.88 in the horizontal, the anterior, and the posterior SCCs. The mean highest coherent frequency was 5.55Hz, 6.52Hz, and 5.59Hz in the horizontal, the anterior, and the posterior SCCs. The significant factors associated with the CR of hearing in the univariate analysis were the normal caloric test ( $p =0.001$), the normal result in vHIT of the posterior SCC ( $p =0.006$), the greater highest coherent frequency of the horizontal SCC ( $p =0.037$) and the posterior SCC ( $p < 0.001$). After adjustment for the other factors using the forward stepwise method, the results remained robust in CR for the greater highest coherent frequency of the posterior SCC (HR + 2.11, 95% CI 1.86-2.35, Table 2). In Pearson correlation analysis in Table 3, the greater highest coherent frequency of the posterior SCC was statistically significantly correlated to the higher VOR gain (0.553), the lower overt saccade percentage (-0.314), and the lower total saccade percentage (-0.257).

**TABLE 1:** Demographic Data and Univariate Analysis of Hearing Outcome, n = 64

Variables		Complete response ( $n =20$)	Partial + No response ( $n =44$)	$p$ value
Gender				0.164
	Male	10 (50%)	14 (31.8%)	
	Female	10 (50%)	30 (68.2%)	
Age				1.000*
	< 65	16 (80%)	34 (77.3%)	
	⪴65	4 (20%)	10 (22.7%)	
Side				0.199
	Left	7 (35%)	23 (52.3%)	
	Right	13 (65%)	21 (47.7%)	
DM				0.739*
	No	17 (85%)	35 (79.5%)	
	Yes	3 (15%)	9 (20.5%)	
CCIS				1.000*
	0–1	17 (85%)	37 (84.1%)	
	⪴2	3 (15%)	7 (15.9%)	
Primary high-dose steroid treatment				0.064*
Intravenous dexamethasone		12 (66.7%)	39 (88.6%)	
Oral prednisolone		6 (33.3%)	5 (11.4%)	
Caloric test				0.001
	Normal	16 (80%)	15 (34.1%)	
	Abnormal	4 (20%)	29 (65.9%)	
c-VEMP				0.199
	Normal	13 (65%)	21 (47.7%)	
	Abnormal	7 (35%)	23 (52.3%)	
o-VEMP				0.115
	Normal	17 (85%)	29 (65.9%)	
	Abnormal	3 (15%)	15 (34.1%)	
vHIT				0.097
	Normal	14 (70%)	21 (47.7%)	
	Abnormal	6 (30%)	23 (52.3%)	
vHIT of the horizontal SCC				0.479
	Normal	18 (90%)	35 (79.5%)	
	Abnormal	2 (10%)	9 (20.5%)	
vHIT of the anterior SCC				1.000*
	Normal	17 (85%)	38 (86.4%)	
	Abnormal	3 (15%)	6 (13.6%)	
vHIT of the posterior SCC				0.006
	Normal	18 (90%)	24 (54.5%)	
	Abnormal	2 (10%)	20 (45.5%)	
VOR gain				
	Horizontal SCC	1.01 ±0.13	0.96 ±0.28	0.333
	Anterior SCC	0.99 ±0.23	0.95 ±0.20	0.399
	Posterior SCC	0.98 ±0.17	0.83 ±0.30	0.055
Total saccade percentage				
	Horizontal SCC	33.35 ±32.11	51.30 ±38.44	0.074
	Anterior SCC	8.15 ±10.67	11.36 ±22.93	0.447
	Posterior SCC	22.4 ±26.36	30.43 ±35.68	0.319
Overt saccade percentage				
	Horizontal SCC	27.60 ±33.58	46.66 ±38.48	0.061
	Anterior SCC	1.00 ±3.08	2.48 ±12.47	0.605
	Posterior SCC	11.40 ±24.17	21.80 ±33.49	0.166
Covert saccade percentage				
	Horizontal SCC	7.60 ±9.99	8.75 ±17.24	0.783
	Anterior SCC	7.65 ±10.32	9.23 ±20.17	0.743
	Posterior SCC	13.00 ±14.87	10.86 ±20.00	0.671
Highest coherent frequency				
	Horizontal SCC	6.30 ±1.56	5.21 ±2.03	0.037
	Anterior SCC	6.94 ±1.04	6.32 ±1.57	0.114
	Posterior SCC	7.00 ±0.87	4.94 ±2.28	< 0.001

DM: diabetes mellitus; CCIS: Charlson comorbidity index score; c-VEMP: cervical vestibular myogenic evoked potentials; o-VEMP: ocular vestibular myogenic evoked potentials; vHIT: video head impulse test; SCC: semicircular canal; VOR: vestibulo-ocular reflex; MSWC: magnitude-squared wavelet coherence

^*^ Fisher’s exact test

**TABLE 2:** Multiple Variate Analysis With Stepwise Forward Method for Hearing Outcome, n = 64

Variables	$p$ value	HR	95% CI
Highest coherent frequency of the posterior SCC	0.002	2.11	1.86–2.35
Normal caloric test	0.082		
Normal vHIT of the posterior SCC	0.418		
Highest coherent frequency of the horizontal SCC	0.573		

MSWC: magnitude-squared wavelet coherence; SCC: semicircular canal; vHIT: video head impulse test

**TABLE 3:** Spearman Correlation Analysis of Magnitude-Squared Wavelet Coherence With Other Variables in the Posterior Semicircular Canal, n = 64

	VOR gain	Total saccade percentage	Overt saccade percentage	Covert saccade percentage
Highest coherent frequency of the posterior SCC	0.553 (< 0.001)	−0.257 (0.04)	−0.314 (0.011)	0.123 (0.332)

MSWC: magnitude-squared wavelet coherence; SCC: semicircular canal

To explore the utility in ANN classification of hearing outcomes (Table 4, 5, and 6), CNN analyses with repeated five-fold cross-validation were done to determine the mean accuracy for (CR vs. PR + NR), (OS of CR vs. PR + NR), (EDE of CR vs. PR + NR), and (ITS of CR vs. PR + NR). In each dataset and cropping style, the feature extraction in CNN with proceeding SVM demonstrated greatly higher accuracy and much lower variability in standard deviation than the pure CNN analysis.

**TABLE 4:** Artificial Neural Network Classification Accuracy of Hearing Outcomes in Horizontal Cropping Style

Dataset		Alexnet+SVM		Resnet+SVM		VGG19+SVM		Alexnet		Resnet		VGG19	
	5fCV	Mean	STD	Mean	STD	Mean	STD	Mean	STD	Mean	STD	Mean	STD
Original	1	82.4	12.5	83.1	11.3	81.4	13.3	82.7	11.5	64.1	9.0	50.3	12.7
	2	81.1	8.1	82.9	8.9	76.4	7.5	70.4	8.7	67.2	10.2	56.4	9.8
	3	76.5	8.5	89.1	3.7	81.1	6.5	76.6	4.5	60.7	9.2	67.0	4.4
	4	81.3	7.8	79.6	6.4	81.4	6.0	71.8	10.6	67.2	3.0	71.8	6.5
	5	81.3	3.6	82.7	3.8	89.1	3.7	73.1	12.9	67.0	9.5	65.6	6.0
	6	79.5	8.6	79.6	8.0	84.2	7.3	76.5	10.9	67.2	5.7	61.0	13.5
	7	82.8	5.7	82.7	6.2	79.9	10.3	74.9	16.6	62.4	3.6	65.2	13.1
	8	77.8	10.2	87.6	6.1	78.2	13.1	79.9	12.4	74.9	6.4	62.6	12.1
	9	84.3	4.9	84.3	4.9	82.8	9.0	73.4	7.7	73.4	10.4	58.0	13.0
	10	79.7	7.7	84.2	5.5	84.0	14.9	68.9	8.0	62.5	7.2	58.9	14.1
	Total	80.7	8.4	83.6	7.4	81.8	10.3	74.8	11.6	66.7	9.0	61.7	12.5
OS	1	90.9	7.5	90.8	5.8	89.7	2.4	79.5	10.9	64.9	7.7	56.3	12.5
	2	89.9	7.3	93.0	6.9	90.8	4.8	81.8	6.7	60.2	8.3	69.2	10.7
	3	88.6	10.2	91.0	5.7	88.5	5.2	87.5	7.5	62.3	9.4	67.1	9.5
	4	88.6	3.8	95.4	5.6	89.7	6.7	83.9	2.5	63.7	13.2	62.5	11.8
	5	89.7	6.9	86.5	5.5	93.2	5.4	84.0	7.0	69.3	5.6	61.1	12.8
	6	88.7	6.9	91.7	9.3	94.4	6.1	78.4	11.5	62.6	8.8	59.1	8.2
	7	89.8	4.1	90.7	7.2	86.3	6.0	78.5	13.4	66.2	10.1	62.7	4.7
	8	93.2	4.1	93.3	5.5	90.8	5.8	83.1	11.5	61.5	7.6	61.4	7.1
	9	93.1	8.4	94.2	5.1	88.5	12.9	79.9	17.7	63.8	9.2	63.4	12.5
	10	92.1	4.5	94.4	6.1	85.2	10.7	90.8	7.8	60.3	11.0	55.6	11.3
	Total	90.5	6.9	92.1	6.8	89.7	7.7	82.7	11.1	63.5	9.7	61.8	11.2
EDE	1	92.0	6.9	88.6	6.3	91.0	6.6	85.1	9.4	61.2	11.5	66.0	10.3
	2	93.3	4.1	89.7	4.3	87.4	5.7	71.5	11.2	68.8	12.7	58.2	12.3
	3	92.0	5.7	89.5	10.2	86.3	2.9	73.8	11.8	67.1	21.1	63.7	14.3
	4	88.7	10.7	89.7	9.3	90.8	4.8	85.4	6.5	65.8	12.3	73.6	11.3
	5	91.0	7.5	89.9	7.3	84.2	8.0	69.4	22.7	58.2	17.2	63.9	11.5
	6	88.6	9.3	88.6	9.9	89.7	6.6	86.4	7.5	64.5	12.6	65.8	8.4
	7	90.9	5.9	85.1	7.7	87.6	11.3	79.4	9.0	58.2	6.3	61.3	10.6
	8	94.2	3.7	88.6	6.1	85.2	4.4	69.4	9.6	62.9	11.8	63.8	8.6
	9	92.2	7.5	89.7	5.5	87.4	4.6	80.9	8.8	61.6	10.3	60.2	5.3
	10	92.1	2.6	89.8	4.1	86.7	9.0	87.5	2.1	65.0	13.1	55.6	12.4
	Total	91.5	7.0	88.9	7.5	87.6	7.2	78.9	13.0	63.3	13.8	63.2	11.8
ITS	1	90.8	4.8	90.8	8.0	88.6	3.8	79.7	12.8	71.4	8.5	51.1	2.9
	2	88.4	8.3	93.3	6.5	81.8	6.5	79.8	11.2	80.4	9.7	74.0	15.3
	3	90.7	8.0	92.0	2.8	87.4	10.0	82.9	5.3	69.2	14.2	66.0	13.8
	4	92.1	5.6	93.2	4.3	86.5	6.5	85.3	8.3	72.6	8.6	66.1	13.4
	5	86.5	7.4	92.1	2.6	86.5	7.5	84.3	11.3	72.7	7.5	54.4	9.1
	6	91.0	2.5	89.9	9.5	84.2	13.4	80.7	10.8	72.6	11.2	58.0	7.3
	7	87.7	13.3	88.6	6.1	83.3	9.9	81.8	6.5	69.3	4.7	55.5	14.0
	8	91.1	5.7	90.9	4.5	84.3	10.6	75.1	10.1	62.6	14.2	65.9	16.9
	9	92.1	6.7	93.0	6.7	86.4	8.2	90.9	2.7	66.9	7.5	62.8	11.3
	10	88.5	6.5	92.0	7.8	84.1	5.5	87.5	6.5	64.9	11.0	63.8	11.6
	Total	89.9	7.6	91.6	6.4	85.3	8.8	82.8	10.0	70.3	11.2	61.8	13.9

SVM: support vector machine; 5fCV: 5-fold cross-validation; STD: standard deviation; OS: over-sampling; EDE: extensive data extraction; ITS: interpolation of time series

**TABLE 5:** Artificial Neural Network Classification Accuracy of Hearing Outcomes in Vertical Cropping Style

		Alexnet+SVM		Resnet+SVM		VGG19+SVM		Alexnet		Resnet		VGG19	
	5fCV	Mean	STD	Mean	STD	Mean	STD	Mean	STD	Mean	STD	Mean	STD
Original	1	79.6	11.7	81.3	12.5	79.9	11.4	64.1	10.2	70.2	3.5	61.1	17.2
	2	81.0	11.1	81.3	6.1	81.1	6.5	70.4	2.3	73.3	6.6	61.4	18.0
	3	81.3	15.0	79.9	16.5	76.8	10.7	67.3	5.2	70.0	18.6	61.0	11.6
	4	78.2	10.1	81.4	7.7	86.0	7.4	73.2	14.9	70.2	3.5	68.7	5.0
	5	81.3	10.4	82.7	6.2	77.8	11.3	69.1	12.7	70.1	11.9	60.7	7.8
	6	89.1	6.1	85.8	9.4	75.0	7.5	71.6	10.0	68.7	1.0	68.7	9.8
	7	78.2	5.5	86.1	10.2	76.6	9.5	70.5	12.0	70.2	6.0	63.9	6.6
	8	84.3	4.9	84.5	12.8	79.6	11.7	76.4	14.1	70.2	7.7	68.7	1.0
	9	78.1	10.3	79.5	9.9	78.2	8.8	69.1	16.7	70.2	7.7	68.7	1.0
	10	78.2	7.3	84.5	12.8	79.6	11.7	60.9	17.6	75.1	11.1	71.9	7.7
	Total	80.9	10.3	82.7	11.1	79.1	10.3	69.3	13.1	70.8	9.3	65.5	11.0
OS	1	89.6	6.9	91.0	7.6	87.4	6.8	70.7	9.0	71.8	14.0	55.7	4.8
	2	87.4	6.8	93.2	5.6	89.7	6.6	66.0	14.7	68.3	8.2	62.8	9.7
	3	87.6	8.1	89.8	4.1	85.3	7.4	78.6	9.3	59.2	6.8	56.7	6.2
	4	88.6	5.1	90.9	2.7	86.4	13.4	80.7	4.6	64.7	16.6	54.9	19.3
	5	88.7	4.8	88.7	3.4	82.9	7.1	66.9	15.2	67.2	10.4	64.7	13.4
	6	92.1	4.4	89.7	5.7	87.5	11.0	77.5	11.4	55.7	6.0	53.3	6.9
	7	93.3	4.1	91.0	7.5	87.4	6.6	80.6	6.9	62.3	13.2	69.2	13.5
	8	85.2	4.4	89.7	6.9	85.3	5.5	64.5	15.8	57.8	8.5	55.6	9.4
	9	90.7	7.2	94.1	4.0	86.4	4.3	76.3	9.3	64.5	13.4	57.9	4.8
	10	90.5	8.5	90.9	4.5	86.5	10.2	80.8	14.5	67.1	4.0	60.3	7.9
	Total	89.4	6.6	90.9	5.7	86.5	8.5	74.2	13.2	63.9	11.8	59.1	11.6
EDE	1	91.0	6.6	85.3	9.6	80.7	9.7	79.4	6.0	60.2	14.6	70.4	9.3
	2	83.0	9.3	88.6	12.1	77.3	3.3	67.1	6.1	56.9	16.6	72.6	17.7
	3	87.5	5.4	86.3	8.6	77.2	8.2	76.2	9.6	57.9	6.1	64.8	11.2
	4	86.5	9.6	87.6	6.3	76.0	8.9	73.8	18.6	55.5	9.8	67.3	14.3
	5	87.6	8.1	88.6	11.1	77.4	5.9	81.6	10.3	57.6	16.3	64.5	17.5
	6	92.0	2.8	87.4	5.9	80.7	8.3	76.1	13.3	61.6	11.9	58.9	7.8
	7	87.4	4.5	93.1	2.5	74.0	12.2	79.2	10.3	60.1	9.7	67.0	8.7
	8	90.8	6.0	86.5	7.6	79.6	5.5	71.3	13.5	66.0	5.6	61.2	12.1
	9	89.8	6.7	89.9	8.1	79.3	8.3	78.1	11.3	56.6	8.9	68.9	20.9
	10	87.4	4.3	93.2	2.1	74.9	12.4	77.2	8.2	69.3	16.4	66.7	11.5
	Total	88.3	7.1	88.6	8.4	77.7	9.0	76.0	12.0	60.2	13.0	66.2	14.3
ITS	1	90.9	2.7	94.3	7.2	85.2	10.3	76.1	8.5	71.6	18.6	78.6	10.6
	2	88.6	6.5	92.0	5.9	84.1	4.1	85.4	6.5	65.9	10.0	64.8	7.9
	3	87.5	5.4	94.4	6.1	85.2	4.8	84.2	9.8	68.2	2.8	77.1	8.7
	4	92.0	4.5	92.2	6.7	82.9	8.2	72.6	13.5	61.2	8.1	73.9	12.5
	5	87.7	5.3	92.1	7.5	82.9	12.4	76.4	14.4	59.2	6.8	72.8	17.1
	6	89.8	9.6	93.2	5.4	78.5	11.2	81.6	12.2	72.8	7.9	65.9	12.2
	7	85.3	7.5	91.0	7.6	80.6	7.0	70.5	11.0	62.5	17.4	66.2	13.2
	8	88.7	6.0	94.4	5.0	86.4	2.5	84.0	4.4	70.7	8.5	69.4	10.1
	9	87.6	8.1	91.9	3.0	84.2	6.3	80.8	7.3	55.6	8.3	64.4	13.1
	10	89.8	7.3	93.1	6.8	78.4	5.7	84.0	8.4	62.5	5.7	54.6	5.4
	Total	88.8	6.8	92.9	6.4	82.8	8.3	79.6	11.3	65.0	11.8	68.8	13.3

SVM: support vector machine; 5fCV: 5-fold cross-validation; STD: standard deviation; OS: over-sampling; EDE: extensive data extraction; ITS: interpolation of time series

**TABLE 6:** Artificial Neural Network Classification Accuracy of Hearing Outcomes in Subtotal Cropping Style

		Alexnet+SVM		Resnet+SVM		VGG19+SVM		Alexnet		Resnet		VGG19	
	5fCV	Mean	STD	Mean	STD	Mean	STD	Mean	STD	Mean	STD	Mean	STD
Original	1	73.4	10.4	75.1	7.2	70.2	6.0	68.2	20.0	57.7	4.9	58.1	19.5
	2	71.8	14.4	79.6	4.1	71.4	15.9	62.7	16.1	66.9	11.3	61.0	15.1
	3	74.7	8.7	81.3	7.8	76.6	10.7	74.9	8.0	71.9	17.2	49.7	16.7
	4	79.7	3.5	79.9	7.7	73.4	7.7	55.9	16.4	70.1	6.8	43.9	11.8
	5	70.4	5.4	79.9	7.7	76.5	4.9	54.6	16.5	65.8	14.0	67.2	3.0
	6	73.7	12.3	78.3	10.1	75.0	10.2	53.4	19.1	59.3	13.2	67.2	3.0
	7	68.9	10.5	81.3	7.8	75.2	11.1	59.1	12.5	62.5	5.3	61.0	15.1
	8	64.1	5.9	75.1	8.7	75.0	13.2	53.3	18.4	65.5	9.6	45.5	15.5
	9	66.9	10.2	82.6	9.8	72.0	10.1	46.7	17.1	68.7	5.0	68.7	1.0
	10	75.0	3.0	81.3	11.5	70.4	11.1	49.7	15.2	64.0	10.7	55.9	16.4
	Total	71.9	10.1	79.4	8.8	73.6	10.8	57.8	18.2	65.2	11.4	57.8	15.9
OS	1	84.0	8.3	94.4	6.1	85.4	7.4	61.1	17.2	44.2	6.4	52.2	2.7
	2	81.5	7.6	89.8	4.1	88.6	9.6	66.9	12.2	50.0	4.7	52.4	2.9
	3	87.4	9.6	91.0	10.3	88.6	7.0	57.9	7.1	53.6	14.0	55.8	10.7
	4	87.5	9.6	87.7	8.1	82.0	13.7	69.7	14.9	54.4	8.0	50.0	1.8
	5	86.4	4.3	86.6	11.4	88.6	9.6	57.9	4.8	54.3	11.0	57.0	10.3
	6	84.1	5.5	87.2	6.5	88.6	3.8	60.5	14.0	60.3	4.6	57.8	8.3
	7	85.5	15.9	88.7	7.2	86.5	10.2	65.3	15.1	64.6	7.3	60.1	12.3
	8	85.1	6.1	88.8	6.0	84.1	4.1	62.5	10.7	49.9	7.4	54.5	3.7
	9	88.6	5.1	92.1	5.6	85.3	7.4	65.8	15.8	46.7	7.1	61.2	9.4
	10	85.2	6.9	86.2	8.5	88.9	7.8	65.0	8.2	56.4	20.7	64.0	13.4
	Total	85.5	8.7	89.2	8.1	86.7	8.8	63.3	13.2	53.4	11.8	56.5	9.6
EDE	1	80.6	7.8	77.4	5.7	78.3	6.6	67.1	11.9	59.4	11.1	58.1	11.0
	2	80.9	7.4	72.4	9.0	77.2	6.2	66.0	8.2	48.8	8.4	52.4	2.9
	3	77.3	5.2	77.4	5.9	81.8	4.4	71.5	13.5	42.3	12.0	55.8	10.7
	4	74.1	8.0	71.7	12.2	75.0	5.8	71.9	14.2	50.8	9.3	50.0	1.8
	5	78.3	4.4	72.9	9.9	80.9	10.1	54.7	10.9	51.2	7.3	57.0	10.3
	6	78.4	6.6	72.8	6.4	77.2	9.5	67.8	19.8	48.7	10.2	57.8	8.3
	7	79.6	7.5	70.5	6.4	79.6	4.2	64.7	15.5	56.7	10.9	60.1	12.3
	8	78.2	9.5	74.0	11.1	71.7	9.1	60.1	15.0	62.6	11.3	54.5	3.7
	9	78.4	6.6	76.2	6.5	83.1	9.1	67.1	7.1	56.8	2.1	61.2	9.4
	10	82.9	6.3	66.4	11.7	71.6	9.1	62.4	10.8	54.2	14.1	64.0	13.4
	Total	78.9	7.4	73.2	9.4	77.7	8.6	65.3	14.1	53.1	11.6	57.1	10.1
ITS	1	80.7	2.9	87.4	4.3	79.6	10.8	79.5	11.6	61.2	12.8	85.2	5.7
	2	79.6	9.5	92.1	4.4	83.1	6.8	84.0	7.0	55.8	15.0	79.8	11.2
	3	83.1	6.0	91.0	4.3	81.6	9.6	73.9	13.4	57.9	7.7	76.0	11.7
	4	73.9	4.2	89.7	2.2	79.4	14.0	83.0	6.0	59.2	11.0	71.9	17.3
	5	79.7	3.9	88.6	6.5	79.4	6.0	80.7	11.5	53.5	16.7	75.2	11.2
	6	80.7	5.8	91.0	7.5	80.9	10.2	83.1	11.5	60.2	5.3	87.5	4.3
	7	83.0	3.5	89.8	4.4	81.8	5.4	79.5	9.2	57.9	7.9	76.5	20.2
	8	83.1	4.7	88.5	5.4	83.0	8.0	64.7	19.4	61.4	14.1	71.9	18.7
	9	81.8	5.4	88.5	5.5	77.3	3.6	80.6	7.9	55.7	6.4	76.7	14.7
	10	80.7	11.4	89.7	4.4	79.7	8.3	74.0	12.6	66.9	7.5	65.5	15.1
	Total	80.6	6.8	89.6	5.2	80.6	8.9	78.3	12.9	59.0	11.7	76.6	15.2

SVM: support vector machine; 5fCV: 5-fold cross-validation; STD: standard deviation; OS: over-sampling; EDE: extensive data extraction; ITS: interpolation of time series

**FIGURE 2.**
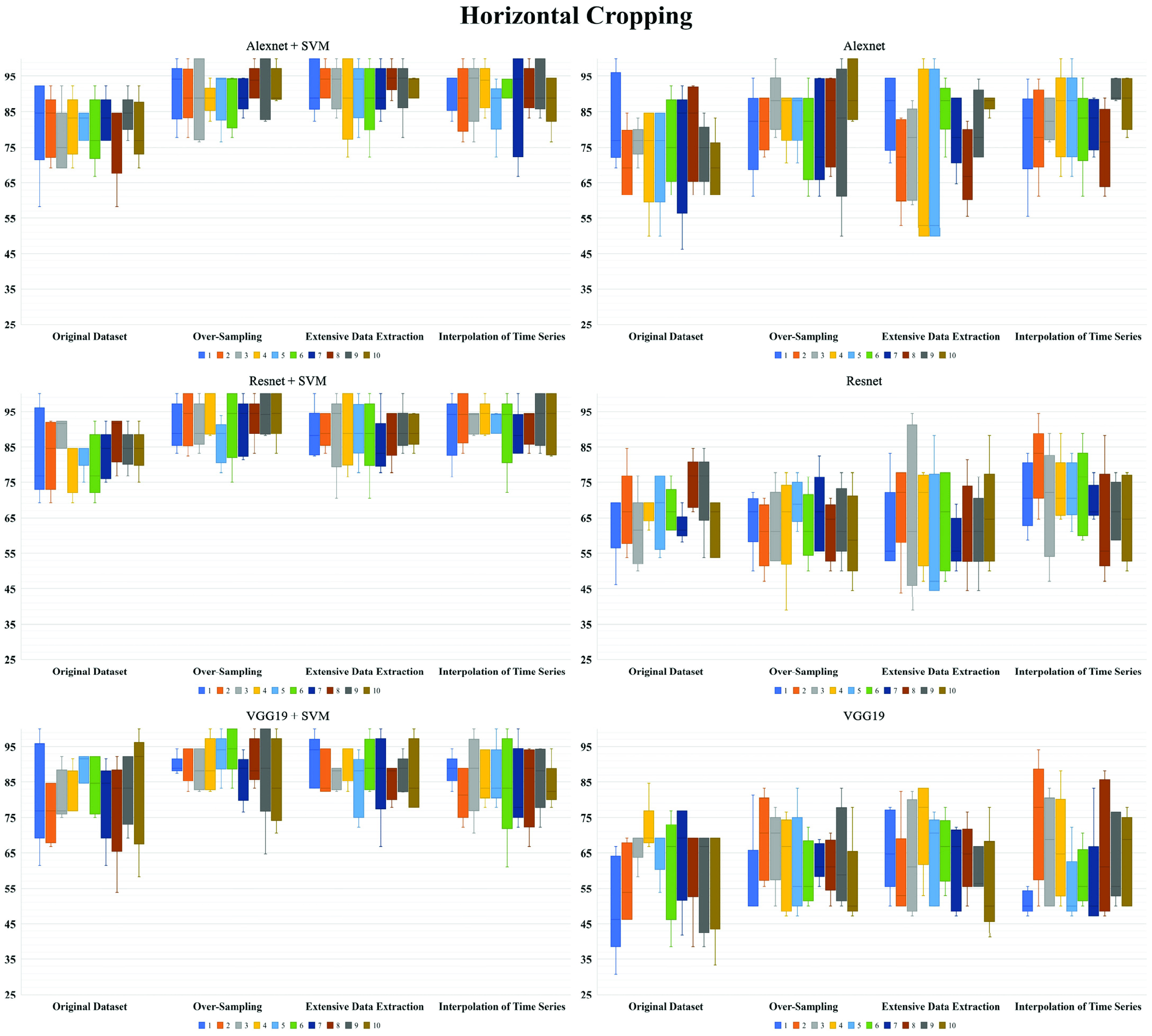
Repeated ten times five-fold cross-validation of hearing outcomes for wavelet coherence plots in horizontal cropping style.

**FIGURE 3.**
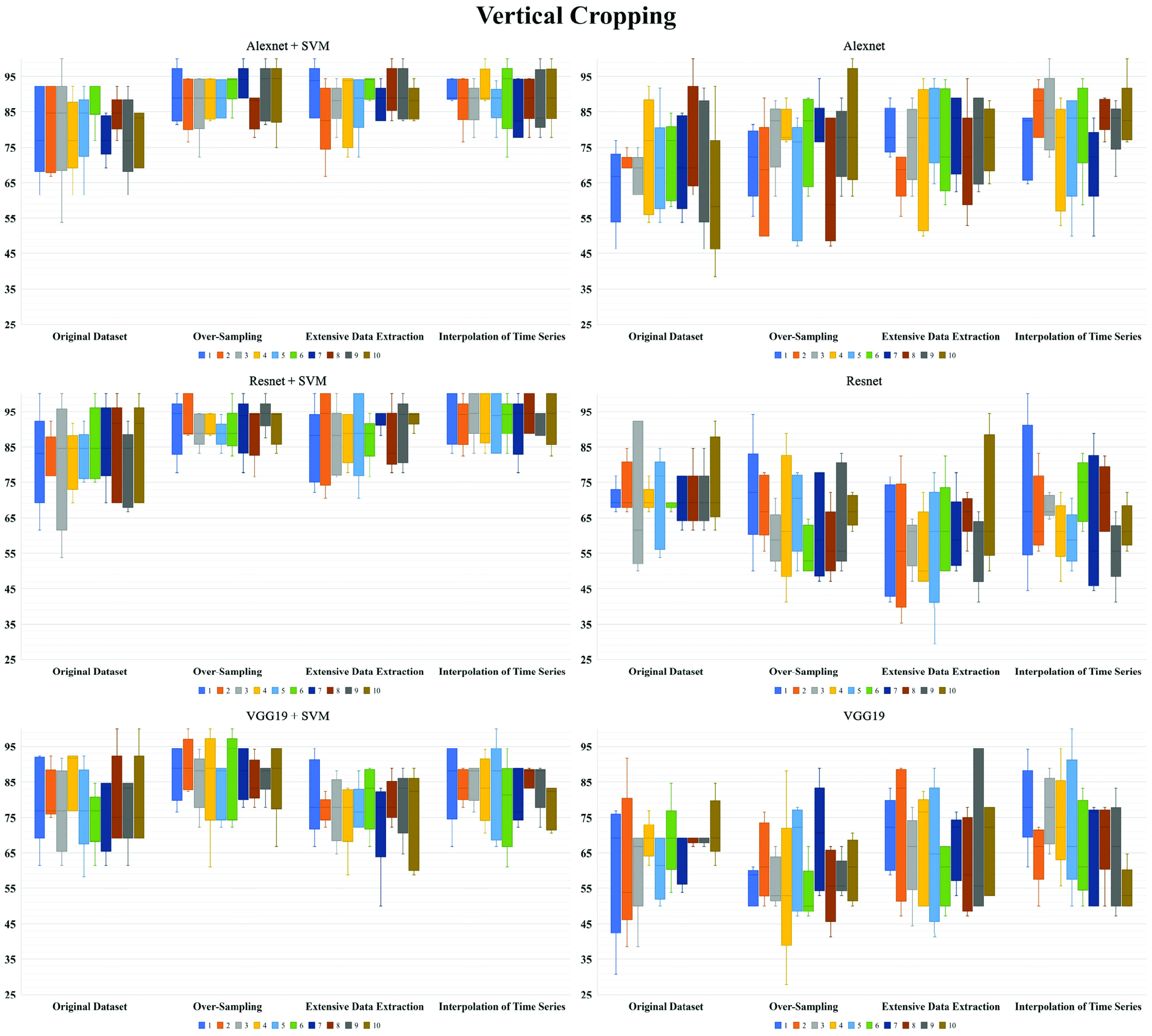
Repeated ten times five-fold cross-validation of hearing outcomes for wavelet coherence plots in vertical cropping style.

Besides, Alexnet showed higher accuracy than Resnet and VGG19 with similar standard deviation in pure CNN analysis, and Resnet + SVM revealed better accuracy than Alexnet + SVM and VGG19 + SVM with similar standard deviation in CNN feature extraction with proceeding SVM classification. In each dataset and ANN models, the horizontal cropping style suggested higher accuracy than vertical and subtotal cropping styles with similar standard deviation. In each ANN model, the augmented datasets had much higher classification accuracy than original datasets in all cropping styles. However, the standard deviations of accuracy in datasets after augmentation showed similar results with original dataset in the horizontal cropping style but generally slightly higher values than original dataset in the vertical and subtotal cropping styles. The best performance of ANN went to the method of feature extraction with Resnet-50 and proceeding SVM in the horizontal image cropping style, the classification accuracy [STD] for (CR vs. PR + NR), (OS of CR vs. PR + NR), (EDE of CR vs. PR + NR), and (ITS of CR vs. PR + NR) were 83.6% [7.4], 92.1% [6.8], 88.9% [7.5], and 91.6% [6.4], respectively. Figure 2, 3, and 4 showed the repeated 5-fold cross-validation results. The feature extraction in AlexNet, ResNet-50, and VGG-19 with proceeding support vector machine (SVM) outperformed as even more stable models to predict hearing outcomes over purely transfer learning of AlexNet, ResNet-50, and VGG-19.

**FIGURE 4.**
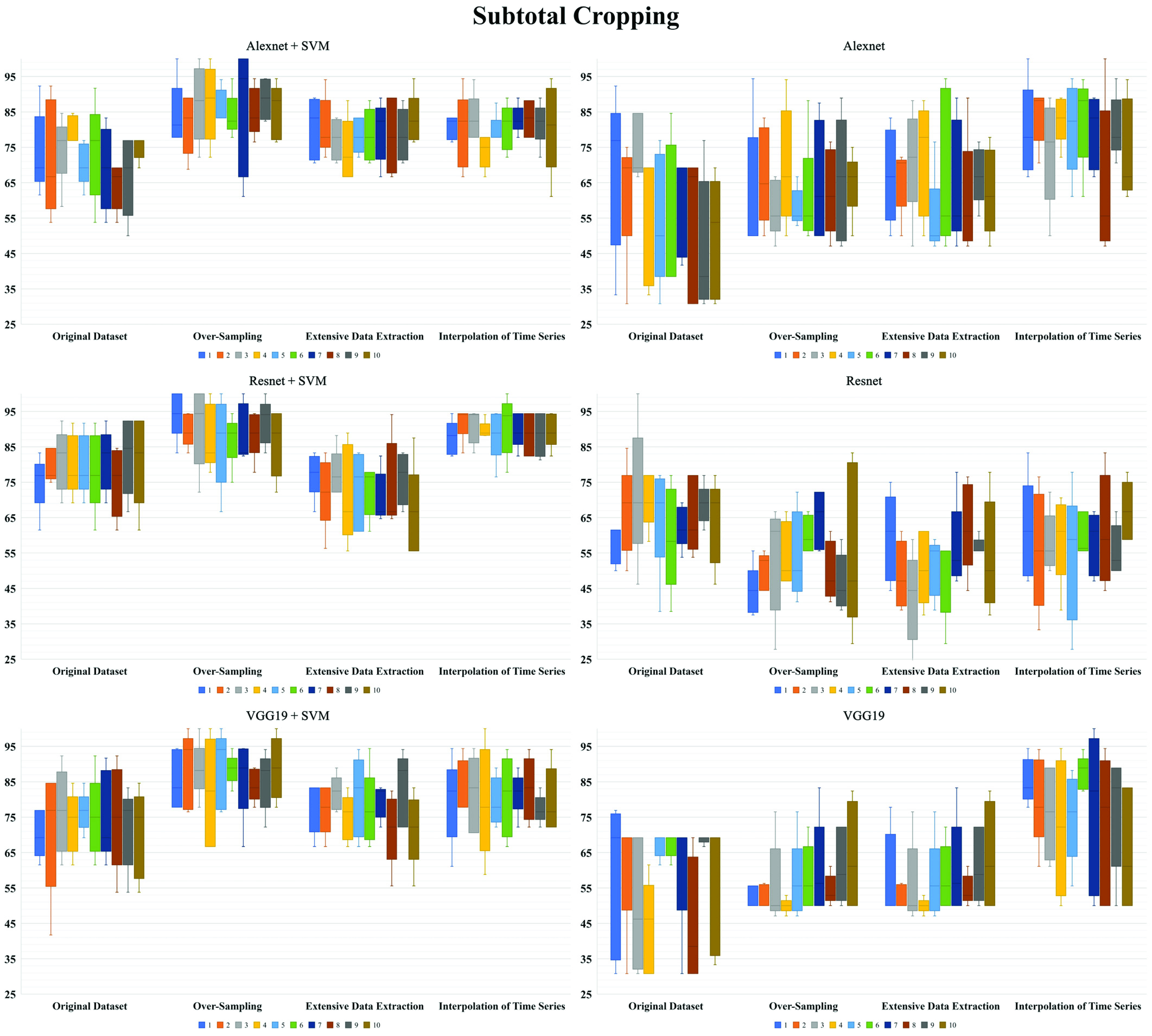
Repeated ten times five-fold cross-validation of hearing outcomes for wavelet coherence plots in subtotal cropping style.

## IV. Discussion

### A. Synopsis of Key Findings

We identified that the great highest coherent frequency was the independent factor associated with CR of hearing in patients with SSNHLV undergoing high-dose steroid treatment. The coherent frequency of vHIT was associated with the responsive eye motion, including VOR gain and the following saccades, to the head impulse with fixed rotation velocity. The comprehensive power spectrum wavelet coherence plot applied for synchrony evaluations in vHIT data were statistically more significant than the time series variables in prognostic predictions. CNN could be utilized to classify WCA, predict treatment outcomes, and facilitate vHIT interpretation. Feature extraction in CNN with proceeding SVM and horizontal cropping style of wavelet coherence plot performed better accuracy and offered more stable model for hearing outcomes in patients with SSNHLV than pure CNN classification.

### B. Strengths of the Study

The main strength of our study was to develop the novel method of the WCA for vHIT, which was more effective in the prediction of hearing outcome due to the precise representation of the overall synchrony of VOR. We recruited the pure cohort of only cases with SSNHLV receiving standard high-dose steroid treatment completing full exams of inner ear battery. With the application of ANN, it appears that this is the first study to apply ANN-assisted coherence analysis to evaluate the hearing outcomes in patients with SSNHLV.

### C. Comparisons With Other Studies

Some previous studies based on the time series analysis of vHIT showed the abnormal vHIT result in the posterior SCC was much higher than in other SCCs and was the significantly negative prognostic factor for incomplete hearing recovery in SSNHLV [11], [12], [13], [29]. In the univariate analysis of our study, the abnormal Caloric test ( $p =0.001$), the abnormal vHIT of the posterior SCC ( $p =0.006$), and the low highest coherent frequencies in the horizontal SCC ( $p =0.037$) and the posterior SCC ( $p < 0.001$) were statistically significantly associated with incomplete hearing recovery in SSNHLV. Moreover, in this first study utilizing the novel application of the WCA in vHIT, our study demonstrated that patients with the greater highest coherent frequency in the posterior SCC had 2.11-times probability of CR of hearing in multivariate analysis, and the coherent frequency of the posterior SCC derived from time-frequency analysis was the only independent factor of CR of hearing. Time series parameters including the abnormal vHIT result in the posterior SCC and the abnormal caloric test were not the significant independent factor for hearing prognosis compared with the parameters resulted from time-frequency analysis. For vHIT, the WCA exerted its great resolution and sensitivity in the interpretation of non-stationary signals.

The highest to the lowest rates of the abnormal vHIT results were 34.4% in the posterior SCC, 17.2% in the horizontal SCC, and 14.1% in the anterior SCC in our cohort. For the patients with incomplete hearing recovery, the VOR gain was much lower in the posterior SCC (0.83 ±0.30) than in the anterior SCC (0.95 ±0.20) and horizontal SCC (0.96 ±0.28), but the total and overt saccades percentages were much higher in the horizontal SCC (51.30 ±38.44 and 46.66 ±38.48) than in the posterior SCC (30.43 ±35.68 and 21.80 ±33.49) and the anterior SCC (11.36 ±22.93 and 2.48 ±12.47). In the vHIT, the vestibulopathy was usually defined by the abnormally low VOR gain and the asymmetry of gains within paired SCCs. However, the significant association between the vestibulopathy and the high percentages of saccades was also noted [30]. Thus, our results showed the higher rates of vestibular end organs damage over the posterior and the horizontal SCCs than over the anterior SCC, and these could be associated with the vascular events of the common cochlear artery and the vestibulocochlear artery perfusion territories [31], [32]. The relationship between the poor VOR function of the posterior SCC and the inferior hearing recovery after steroid treatment could be explained by the most distal blood supply of the posterior SCC by the posterior vestibular artery and its poor collateral perfusions [12]. Furthermore, in the patients with incomplete hearing recovery, the highest coherent frequency in the posterior SCC (4.94 ±2.28) and the horizontal SCC (5.21 ±2.03) were much lower than in the anterior SCC (6.32 ±1.57). The results of WCA were comparable to the abnormal VOR gain of the posterior SCC and the high total percentages of saccades over the horizontal SCC. Based on frequency-dynamic character of VOR, WCA may be more delicately to detect the damage of VOR function of the posterior SCC rather than time series analysis [33].

**FIGURE 5.**
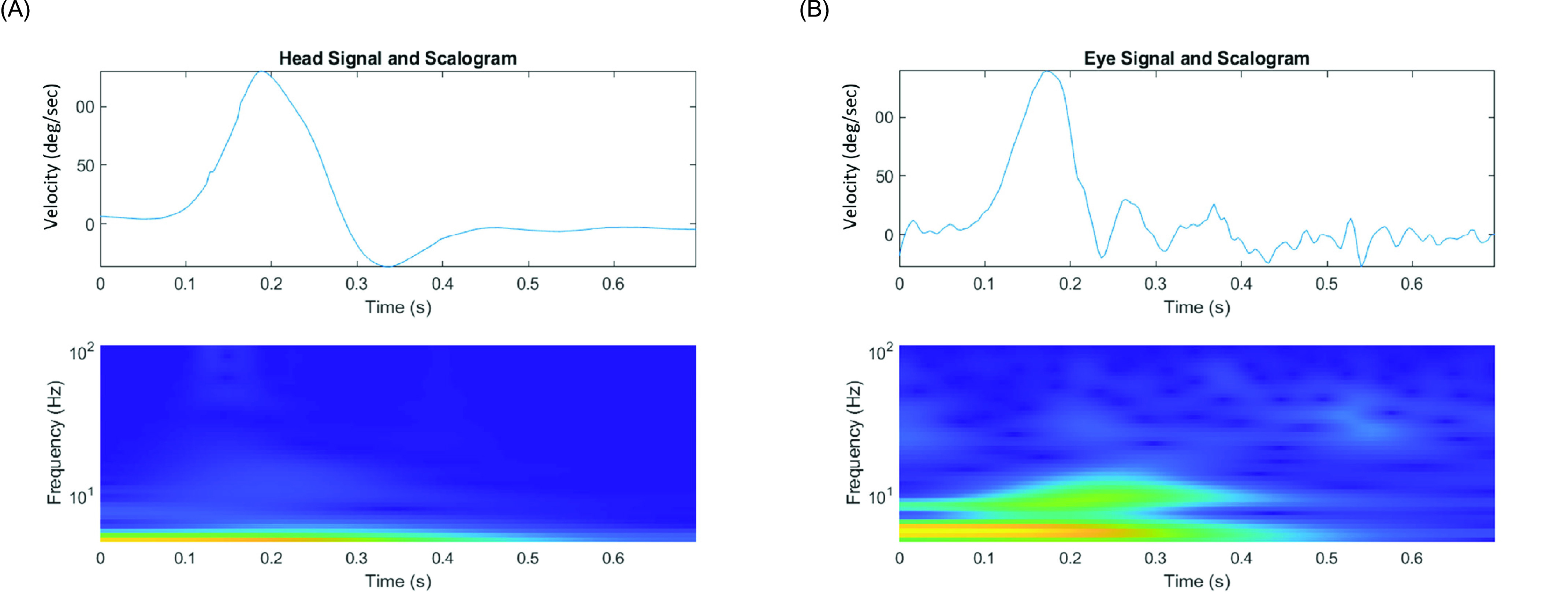
Time-frequency plots and scalograms of the (A) head and (B) eye impulses with poor coherence (3.28) but normal vestibulo-ocular reflex gain (0.78).

The Spearman correlation analysis with parameters in time-frequency analysis and time series analysis was performed to discover the clinical meaning of the parameters in WCA, which showed the great highest coherent frequency in the posterior SCC was statistically correlated to the high VOR gain, the low overt saccade percentage, and the low total saccade percentage. The MSWC was an implement of signal processing to measure the correlation between the head velocity resulted from passive rotation and the corresponding eye velocity in their time-frequency domain. During wavelet transform of vHIT, not merely VOR gain but also saccades may alter the time-frequency plot for eye movement. The WCA took advantage in comprehensive comparison of these sensitive responses of the time-frequency plots between eye and head movement, and the MSWC 0.9 further presented the significantly linear dependance between the paired signals of non-stationary impulses [34]. To clarify the advantages of WCA of vHIT over traditional analysis of VOR gain with an example, Figure 5 illustrated the time series plots and time-frequency plots with scalograms of head and eye velocities from a patient with NR of hearing outcome who had normal VOR gain (0.78), several saccades, and evidently lower highest coherent frequency (3.28) in the posterior SCC. In the time series plots, the head and eye impulses had similar highest rotational velocities, so the normal VOR gain was noted. However, many saccades in time series plot of eye were recorded. Since the frequency of head rotation ranged about 4-8Hz, the highest coherent frequency could evaluate the function of VOR more all-inclusive than time series parameters if the frequency of responsive eye motion was altered by not only VOR gain but also corrective saccades.

In ANN analysis, we found the mean accuracy was higher in the way of feature extraction in CNN and proceeding SVM than pure CNN classification. The highest average accuracy [STD] for (CR vs. PR + NR), (OS of CR vs. PR + NR), (EDE of CR vs. PR + NR), and (ITS of CR vs. PR + NR) were 83.6% [7.4], 92.1% [6.8], 88.9% [7.5], and 91.6% [6.4], respectively, with the feature extraction with Resnet-50 and proceeding SVM in the horizontal image cropping style. However, the best performance on accuracy [STD] of pure CNN classification was only 74.8% [11.6], 82.7% [11.1], 78.9% [13.0], and 82.8% [10.0] for (CR vs. PR + NR), (OS of CR vs. PR + NR), (EDE of CR vs. PR + NR), and (ITS of CR vs. PR + NR) with Alexnet in the horizontal image cropping style. The accuracy variability of standard deviation was much more stable in CNN feature extraction with proceeding SVM classification than pure CNN analysis. Since we used image tiling to comprise the WCAs in all SCCs, the pure CNN structure was quite sensitive to differences of pixel intensity among regional areas in nature images, which may lead to misclassifications and unstable performance of model in tiled images [35]. Besides, among different styles of image cropping, the mean accuracy was higher in the horizontal image cropping style with much more stable variability of accuracy than in the vertical style and then the subtotal style, sequentially. The medians of highest coherent frequency were 5.94Hz, 6.73Hz, and 6.26Hz for the horizontal, anterior, and posterior SCCs in our cohort. The overt and covert saccades after passive head motions were probably associated with the recovery of VOR function [7]. From the point of view of the principal component of analysis, compared with other image cropping styles, the horizontal image cropping style reserved not only the sharp edge of the highest frequency in the narrower frequency window (4-8Hz) but also the information of corrective saccades in more sufficient time window (0.25s after a head motion) without pixel compression after image tiling.

### D. Weaknesses of the Study and Future Work

There were some limitations in the current study. First and foremost, this was a retrospective study using pretrained ANN in a single institute with limited case numbers. For balanced classes and robust feature extractions, more subjects and an ANN originally designed should be adopted in the future to obtain more accurate results of classification and more stable performance of ANN models. Second, although the WCA of vHIT data could better evaluate VOR function and predict hearing prognosis in the patients with SSNHLV, the exact pathophysiology and the hypothesis of vascular insults related to poor hearing recovery and abnormal function of the posterior SCC may need further investigations in evidence of radiology or pathology. Last but not least, for clinical application, the cut-off value of the highest coherent frequency associated with complete recovery of hearing could be evaluated in the future with greater cohort.

## V. Conclusion

The high coherent frequency was a significant independent factor that was associated with good hearing prognosis in the patients who have SSNHLV. WCA demonstrated comprehensive ability in VOR function evaluation and was more robust than time series variables in prognostic predictions. CNN could be utilized to classify WCA, predict treatment outcomes, and facilitate vHIT interpretation. Feature extraction in CNN with proceeding SVM and horizontal cropping style of wavelet coherence plot performed better accuracy and offered more stable model for hearing outcomes in patients with SSNHLV than pure CNN classification.
